# Fluorescent tagging of endogenous IRS2 with an auxin-dependent degron to assess dynamic intracellular localization and function

**DOI:** 10.1016/j.jbc.2024.107796

**Published:** 2024-09-19

**Authors:** Minjeong Jo, Ji-Sun Lee, Claire E. Tocheny, Michael W. Lero, Quyen Thu Bui, Jennifer S. Morgan, Leslie M. Shaw

**Affiliations:** Department of Molecular, Cell & Cancer Biology, University of Massachusetts Chan Medical School, Worcester, Massachusetts, USA

**Keywords:** insulin receptor substrate-2 (IRS2), IGF-1R, CRISPR/Cas9, auxin inducible degron, fluorescence imaging, nuclear translocation

## Abstract

Insulin Receptor Substrate 2 (IRS2) is a signaling adaptor protein for the insulin (IR) and Insulin-like Growth Factor-1 (IGF-1R) receptors. In breast cancer, IRS2 contributes to both the initiation of primary tumor growth and the establishment of secondary metastases through regulation of cancer stem cell (CSC) function and invasion. However, how IRS2 mediates its diverse functions is not well understood. We used CRISPR/Cas9-mediated gene editing to modify endogenous IRS2 to study the expression, localization, and function of this adaptor protein. A cassette containing an auxin-inducible degradation (AID) sequence, 3x-FLAG tag, and mNeon-green was introduced at the N-terminus of the IRS2 protein to provide rapid and reversible control of IRS2 protein degradation and analysis of endogenous IRS2 expression and localization. Live fluorescence imaging of these cells revealed that IRS2 shuttles between the cytoplasm and nucleus in response to growth regulatory signals in a PI3K-dependent manner. Inhibition of nuclear export or deletion of a putative nuclear export sequence in the C-terminal tail promotes nuclear retention of IRS2, implicating nuclear export in the mechanism by which IRS2 intracellular localization is regulated. Moreover, the acute induction of IRS2 degradation reduces tumor cell invasion, demonstrating the potential for therapeutic targeting of this adaptor protein. Our data highlight the value of our model of endogenously tagged IRS2 as a tool to study IRS2 localization and function.

The Insulin Receptor Substrate (IRS) adaptor proteins IRS1 and IRS2 play essential roles in regulating the response of breast and other tumor cells to signaling through the insulin (IR) and Insulin-like Growth Factor-1 (IGF-1R) receptors, as well as to some integrin and cytokine receptors (1, 2). Both IRS1 and IRS2 are expressed in breast tumors, but their expression patterns and functions differ in a subtype-dependent manner. IRS1 expression is highest in well-differentiated ER + luminal breast tumors, and expression decreases as tumors become more poorly differentiated (3, 4). IRS1 has been implicated in regulating proliferation and survival in response to insulin and IGF-1 signaling (IIS) (5, 6, 7). In contrast, IRS2 expression is elevated in more aggressive subtypes such as Basal/Triple Negative Breast Cancer (TNBC), and the membrane-localized expression of IRS2 is associated with reduced overall survival of breast cancer patients (8, 9). Irs2 contributes to both the initiation of primary tumor growth and the establishment of secondary metastases through regulation of CSC function and invasion in response to IIS (1, 10, 11, 12). In contrast, loss of Irs1 expression enhances metastatic rates (13). Together, studies to date highlight the divergent functions of the IRS adaptor proteins in breast cancer. However, how each adaptor protein regulates diverse functions is not well understood, and more detailed studies are required to address this lack of mechanistic insight.

Much of the understanding about how the IRS proteins impact tumor cell functions has come from studies of cell lines in which IRS expression has been stably knocked down by shRNA or knocked out by Cre/lox recombination or CRISPR/Cas9 mediated gene targeting and restoration of exogenously expressed WT and mutant proteins (1, 10, 11, 12). These studies have provided important information about these proteins, but a rigorous understanding of endogenous protein localization in live cells and the functional impact of acute loss of protein expression is lacking. CRISPR-mediated endogenous gene tagging provides the potential to explore protein function and subcellular localization in living cells or more complex model systems. For example, fluorescently tagged proteins are detectable in live cells enabling a complete and real-time investigation of their subcellular location (14). In addition, the auxin-inducible degron (AID) system has provided a unique tool for rapid and reversible depletion of a target protein, controlled by the addition of an auxin molecule (indole-3-acetic acid, 3-IAA) (15, 16). When an AID is fused to a target protein and an auxin receptor F-box protein is exogenously expressed, a SKP1-CUL1-F-Box (SCF) ubiquitin E3 ligase complex is formed in eukaryotic cells using endogenous components. Following the addition of 3-IAA, polyubiquitination and rapid proteasomal degradation of the degron-fused protein occur rapidly. Acute loss-of-function experiments can reveal significant insights into molecular pathways and gene function, and the AID system has been successfully applied in many cell lines and organisms (17).

In this study, we utilized CRISPR/Cas9-mediated homology-directed repair to generate cells expressing endogenous IRS2 containing an N-terminal auxin-inducible degron and mNeon-green fluorescent protein to monitor IRS2 expression and function. The resources developed in this study should be of value in studying the complexity of IRS2 function and dynamic regulation, as well as the potential of IRS2 as a therapeutic target in cancer.

## Results

### CRISPR/Cas9-mediated targeting of an AID-FLAG-mNeon-green tag at the IRS2 N-terminus

**Figure 1:**
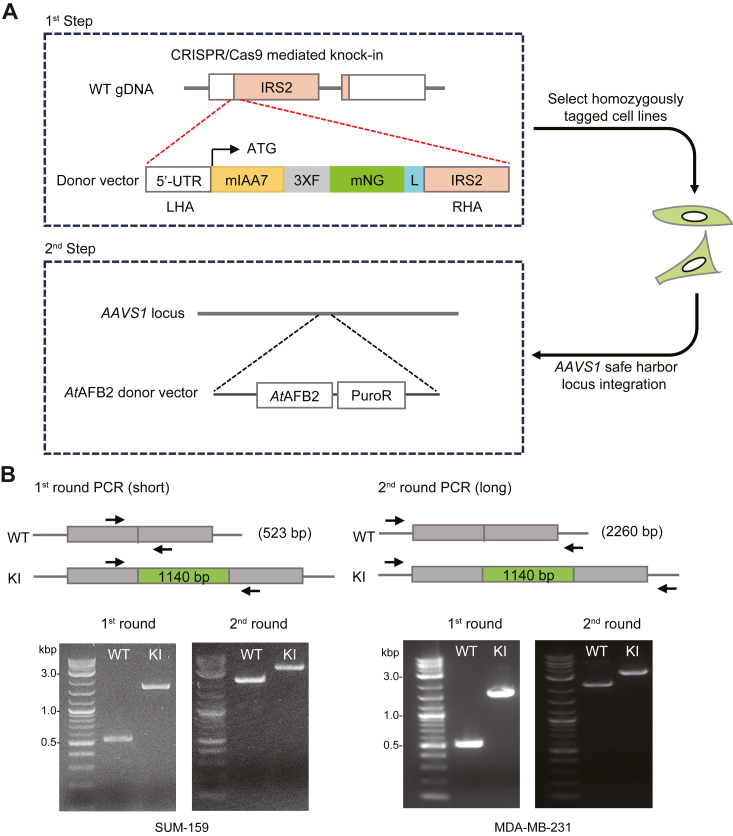
**Schematic of the generation of IRS2 knock-in cells.***A*, targeted integration of AID (mIAA7), FLAG (3XF), and mNeon-green (mNG) tags with linker (L) into the N-terminus of IRS2 and integration of *At*AFB2 and the PuroR cassette into the *AAVS1* safe harbor locus in homozygously tagged clones by selection with puromycin. LHA, left homology arm; RHA, right homology arm. *B*, PCR genotyping strategy for selecting homozygously tagged *DFN-IRS2*. First-round primers complementary to the modified loci were used to amplify wild-type alleles (523 bp) and knock-in alleles (1663 bp). Second-round primers complementary to *IRS2* outside the donor plasmid homology arms were used in long-distance PCR for wild-type alleles (2260 bp) and knock-in alleles (3400 bp). Indicated amplicon sizes confirm the homozygous integration of the tags.

To facilitate real-time analysis of endogenous IRS2 expression and localization and study the impact of acute loss of IRS2 expression on cellular function, we utilized CRISPR/Cas9-mediated homology-directed repair (HDR) to insert a cassette that includes an auxin-inducible Degron (AID) sequence from *Arabidopsis thaliana* IAA17 (mIAA7 degron), a 3x-FLAG tag and the fluorescent mNeon-green protein (DFN-cassette) at the N-terminus of the IRS2 protein (Fig. 1*A*). A flexible linker was added between this cassette and the N-terminus of IRS2 to prevent interference with IRS2 function. Guide RNAs that recognized protospacer adjacent motif (PAM) sequences within 20 bp of the start codon of *IRS2* were screened using Tracking of Indels by Decomposition (TIDE) analysis to identify the guide with the most efficient cutting (Fig. S1*A*).

DFN knock-in (KI) (DFN-IRS2) cells were generated in two steps using CRISPR/Cas9-mediated HDR. In the first step, SUM-159 and MDA-MB-231 triple-negative breast carcinoma cells were electroporated with the most efficient gRNA complexed with Cas9 protein, and a donor fragment containing the DFN-cassette with left and right homology regions corresponding to the *IRS2* genomic sequence (Fig. 1*A*). After electroporation, cells were plated and allowed to recover for 24 to 48 h before sorting mNeon-green positive cells into 96-well plates (Fig. S1*B*). Expanded clones were screened by genomic PCR to confirm the knock-in of the DFN cassette into the N-terminus of the IRS2 protein. In the first round of PCR screening, primers amplified the *DFN* cassette alone and in the second round, primers amplified the *DFN* cassette and both homology arms (Fig. 1*B*). A single band with an increased shift of 1140 bp, the size of the DFN cassette, in the KI cells indicates homozygous expression of *DFN-IRS2*, which was confirmed by Sanger sequencing. In the second step, selected single homozygously-tagged clones were modified to stably express the *A. thaliana* F-box protein AFB2 (AtAFB2), which upon treatment with auxin, binds to the mIAA7 degron and promotes ubiquitination and degradation of tagged proteins (17). AtAFB2 was inserted into the *AAVS1* safe harbor locus by CRISPR/Cas9-mediated HDR (Fig. 1*A*) (16, 17). After selection in puromycin, cells were treated with Auxin for 1 h and then sorted for loss of mNeon-green fluorescence to further enrich for *At*AFB2 positive populations.

### Characterization of cells expressing endogenously tagged IRS2

**Figure 2:**
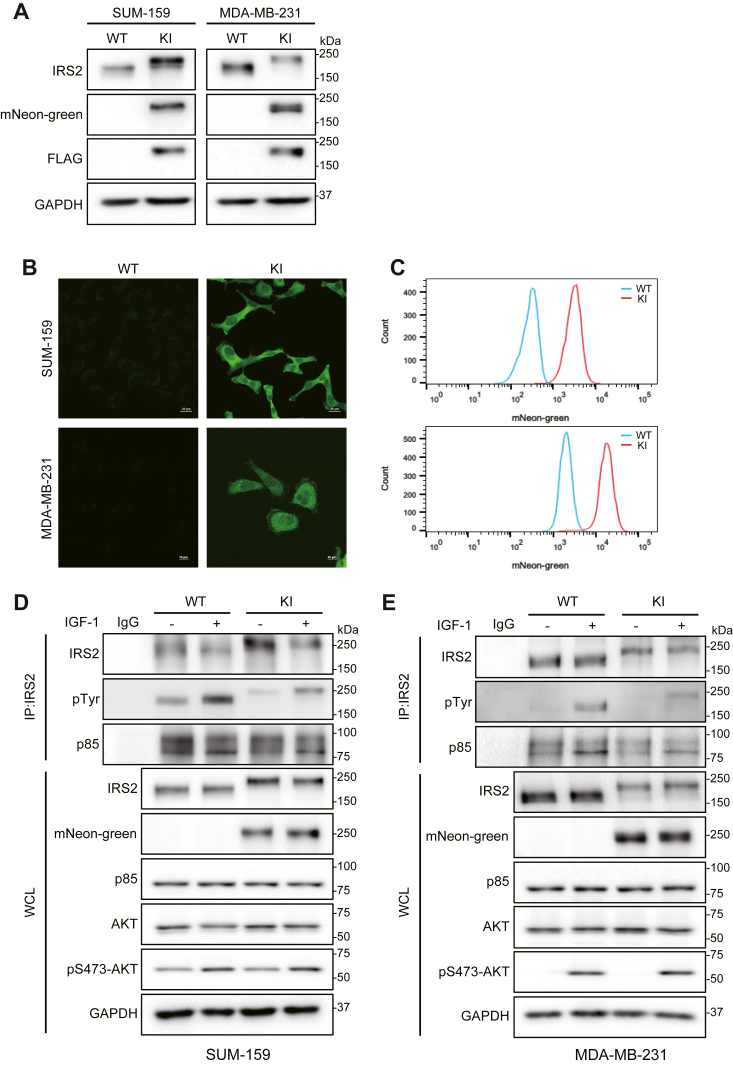
**Characterization of DFN-IRS2 protein expression and function.***A*, cell extracts from parental (WT) and DFN (KI) SUM-159 and MDA-MB-231 cells were immunoblotted with antibodies that detect IRS2, mNeon-green and FLAG. *B*, fluorescence imaging of parental (WT) and DFN-IRS2 (KI) SUM-159 and MDA-MB-231 cells. Scale bars = 25 μm (SUM-159) or 10 μm (MDA-MB-231). *C*, flow cytometric analysis of parental (WT) and DFN-IRS2 (KI) SUM-159 and MDA-MB-231 cells. *D and E*, parental (WT) and DFN-IRS2 (KI) SUM-159 and MDA-MB-231 cells were serum starved and stimulated with IGF-1 (50 ng/ml) for 10 min. Aliquots of cell extracts that contained equivalent amounts of total protein were immunoprecipitated with IRS2-specific antibodies and the immune complexes were immunoblotted with antibodies that recognize either IRS2, phosphotyrosine (pTyr), or p85. Aliquots of cell extracts that contained equivalent amounts of total protein (WCL) were immunoblotted with the indicated antibodies.

Cell extracts were screened by immunoblotting to confirm the expression of the DFN-IRS2-tagged protein. Insertion of the DFN cassette resulted in the expected mobility shift of 42 kDa for the IRS2 protein, which was also recognized by FLAG and mNeon-green specific antibodies (Fig. 2*A*). mNeon-green expression in the KI cells was also confirmed by fluorescence imaging and flow cytometry (Fig. 2, *B* and *C*). Parental cells expressing WT-IRS2 were negative for mNeon-green and FLAG expression by both immunoblot and imaging.

IRS2 is recruited to the phosphorylated cytoplasmic tails of the insulin and IGF-1 receptors upon stimulation with insulin/IGF-1 (18). Upon binding, IRS2 undergoes tyrosine phosphorylation by the intrinsic receptor tyrosine kinases, creating new docking sites for downstream signaling effectors such as PI3K (19). To confirm that the addition of the DFN cassette to the N-terminus of IRS2 does not interfere with this signaling function, cells expressing WT-IRS2 and DFN-IRS2 were stimulated with IGF-1 (50 ng/ml) and cell extracts were immunoprecipitated with IRS2 antibodies. Immune complexes were resolved by SDS-PAGE and immunoblotted with phosphotyrosine (pTyr) and PI3K (p85)-specific antibodies. Increased phosphorylation and binding to PI3K were observed after stimulation with IGF-1 for WT-IRS2 and DFN-IRS2 in both SUM-159 and MDA-MB-231 cells (Fig. 2, *D* and *E*). Activation of AKT downstream of PI3K, as measured by phosphorylation of S473-AKT, was also observed for both KI cell lines.

### Live imaging of DFN-IRS2 reveals dynamic intracellular localization

**Figure 3:**
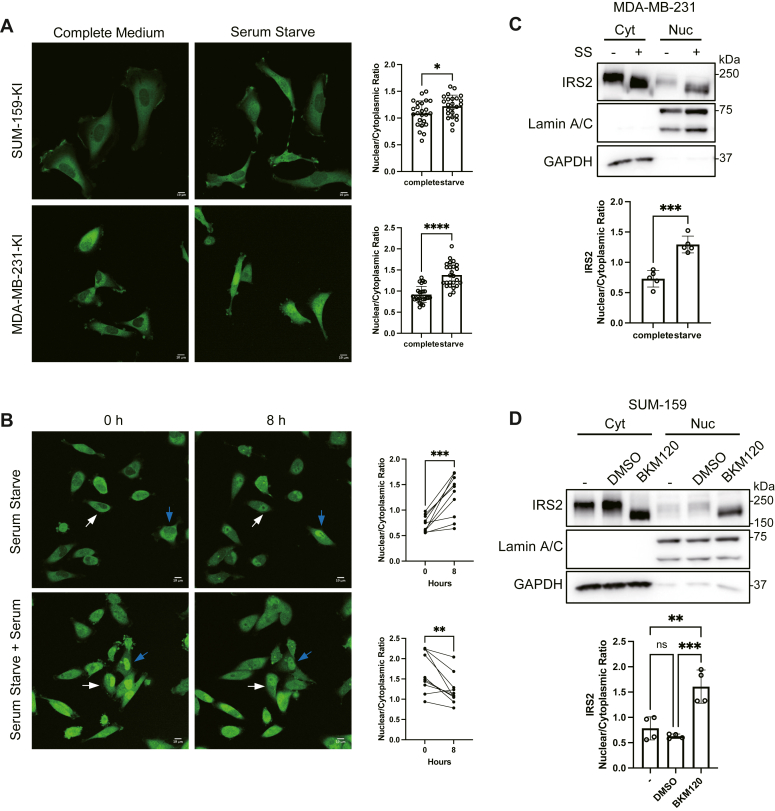
**Analysis of DFN-IRS2 intracellular localization by live imaging.***A*, DFN-IRS2 (KI) SUM-159, and MDA-MB-231 cells were analyzed by live fluorescence imaging in a complete culture medium or culture medium lacking FBS (serum starve). The data shown in the graphs on the right represent the mean ± S.D. of the nuclear/cytoplasmic ratio of DFN-IRS2 (n = 24) from a representative experiment. Scale bars = 10 μm. *B*, DFN-IRS2 KI MDA-MB-231 cells were imaged for 8 h in complete culture medium or culture medium lacking FBS (serum starve) for 8 h. Top row, cells began in full culture medium and were serum starved for 8 h. Bottom row, cells began under serum-starved conditions and were incubated in complete culture medium for 8 h. The images shown capture the start and end of the imaging period. The data shown in the graphs represent the nuclear/cytoplasmic ratio of DFN-IRS2 in individual cells at the beginning and end of the imaging (n = 10). Colored arrows track individual cells over time. Scale bars = 10 μm. *C*, Parental MDA-MB-231 cells were grown in a normal culture medium or culture medium lacking FBS (SS) and extracted to isolate cytosolic and nuclear fractions. Aliquots of cell extracts that contained equivalent amounts of total protein were immunoblotted with the indicated antibodies. The data shown represent the mean ± S.D. of the nuclear/cytoplasmic ratio from five independent experiments. *D*, parental SUM-159 cells were grown in a normal culture medium in the absence (−) or presence of BKM120 or vehicle (DMSO) and extracted to isolate cytosolic and nuclear fractions. Aliquots of cell extracts that contained equivalent amounts of total protein were immunoblotted with the indicated antibodies. The data shown represent the mean ± S.D. of the nuclear/cytoplasmic ratio from four independent experiments. ∗*p* < 0.05, ∗∗*p* < 0.01, ∗∗∗*p* < 0.001, ∗∗∗∗*p* < 0.0001.

Knocking mNeon-green into the N-terminus of IRS2 allows for the analysis of endogenous IRS2 localization in live cells. This analysis eliminates the potential for artifacts from fixation and Ab immunostaining. We assessed DFN-IRS2 expression in live cells under normal growth conditions or after serum starvation. As we had observed previously for WT-IRS2 in fixed cells and in FFPE sections of human tumors, in normal growth medium IRS2 is primarily cytoplasmic with little expression in the nucleus (Fig. 3*A*) (8, 20). However, when cells were serum-starved for 2 h, the nuclear localization of DFN-IRS2 increased modestly in SUM-159 cells and more significantly in MDA-MB-231 cells (Fig. 3*A*). To evaluate the change in DFN-IRS2 localization in MDA-MB-231 cells over time, images were collected hourly for 8 h, and the relative expression of DFN-IRS2 in the nucleus of individual cells was assessed over time. DFN-IRS2 expression in the nucleus increased upon removal of serum and decreased when serum was restored to serum-deprived cells over the 8-h time period (Fig. 3*B* and Movies #1 and #2).

To validate the cytoplasmic and nuclear shuttling of WT-IRS2, cytosolic and nuclear fractions were isolated from parental cells treated under similar serum stimulation or starvation conditions and evaluated by immunoblot. This analysis confirmed that the majority of WT-IRS2 is cytosolic under normal culture conditions and nuclear localization increases significantly upon serum starvation in MDA-MB-231 cells (Fig. 3*C*). However, as we had observed for the imaging of DFN-IRS2 in SUM-159 cells, WT-IRS2 expression did not increase significantly in the nuclear extracts upon serum starvation in parental SUM-159 cells. We hypothesized that the genetic background of the cell lines could impact IRS2 nucleo-cytoplasmic shuttling. SUM-159 cells, but not MDA-MB-231 cells, express an oncogenic PI3K mutation (PIK3CA-H1047L) and the PI3K signaling pathway is constitutively active in these cells as evidenced by the persistence of AKT phosphorylation in serum-starved cells (Fig. 2*D*). To test the involvement of PI3K in the regulation of IRS2 intracellular localization, parental SUM-159 cells were treated with a PI3K inhibitor (BKM120) for 3 h prior to the collection of cytoplasmic and nuclear extracts. Inhibition of PI3K significantly increased the ratio of WT-IRS2 in the nucleus, similar to the increase observed for serum-starved MDA-MB-231 cells (Fig. 3, *C* and *D*).

**Figure 4:**
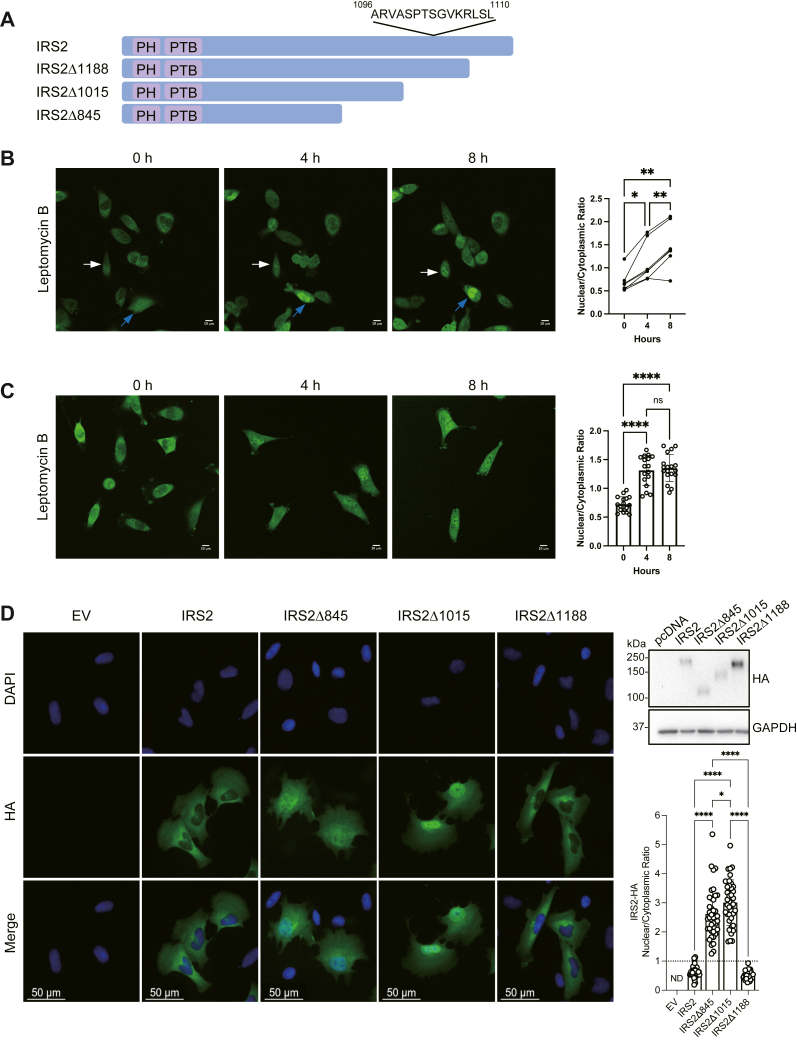
**Regulation of IRS2 intracellular localization.***A*, schematic of WT-IRS2 and IRS2-deletion mutants showing a putative nuclear export signal (NES). *B*, DFN-IRS2 (KI) MDA-MB-231 cells were imaged in a complete culture medium in the presence or absence of Leptomycin B over 8 h, and images from specific time points are shown. The data in the graphs represent the nuclear/cytoplasmic ratio of DFN-IRS2 at each time point in individual cells (n = 7) from a representative experiment. Colored arrows track individual cells over time. Scale bars = 10 μm. *C*, MDA-MB-231 DFN-IRS2 KI cells were imaged in a complete culture medium in the presence or absence of Leptomycin B for 8 h. Representative images from 0, 4, and 8 h are shown. The data in the graphs represent the mean ± S.D. of the nuclear/cytoplasmic ratio of DFN-IRS2 at each time point (n = 17). Scale bars = 10 μm. *D*, SUM-159 cells transfected with HA-tagged WT-IRS2 and IRS2 deletion mutants (shown in A) were fixed and stained with HA antibodies (green) and DAPI (blue). The data shown in the graph represent the mean ± S.D. of IRS2 expression in the nucleus from a representative experiment performed three independent times (n = 40–43 cells). Scale bars = 50 μm. Top right, immunoblot of exogenous IRS2 expression. ∗*p* < 0.05, ∗∗*p* < 0.01, ∗∗∗∗*p* < 0.0001.

Nucleo-cytoplasmic shuttling can be controlled through the regulation of nuclear import or nuclear export. To understand how IRS2 nuclear localization is controlled, we analyzed the sequence of IRS2 to identify putative nuclear import and export sequences. Although we did not find a predicted nuclear import sequence, the sequences between 1096 to 1110 of IRS2 scored highly using the LocNES prediction program that identifies putative CRM1-dependent nuclear export signals (NES) (Fig. 4*A*) (21). To initially assess if IRS2 nuclear localization is controlled by nuclear export regulation, DFN-IRS2 was imaged in MDA-MB-231 cells over 8 h in the presence of the nuclear export inhibitor Leptomycin B. Analysis of DFN-IRS2 localization within individual cells before and after treatment revealed that IRS2 nuclear localization increased over this time period when nuclear export was inhibited (Fig. 4*B* and Movie #3), with maximal nuclear accumulation occurring within 4 h when a larger number of cells were analyzed at each time point (Fig. 4*C*).

To assess if the predicted NES of IRS2 is required for nuclear export, we analyzed a series of C-terminal tail deletions of IRS2 (Fig. 4*A*) for their localization in SUM-159 cells cultured in normal growth medium, which promotes cytosolic localization (Fig. 4*D*). IRS2 lacking the final 150 amino acids (IRS2Δ1188) remained primarily in the cytoplasm, similar to WT-IRS2. However, upon deletion of an additional 173 amino acids (IRS2Δ1015) or 343 amino acids (IRS2Δ845), both of which result in the loss of the putative NES, a significant accumulation of IRS2 expression within the nucleus was observed. Taken together, our data provide evidence that IRS2 nuclear-cytoplasmic localization is regulated at the level of nuclear export and that sequences within the region of IRS2 that contain a putative NES (amino acids 1015-1188) are required for efficient export of IRS2 from the nucleus and maintenance of the predominantly cytosolic localization.

### Reversible IRS2 protein degradation by auxin

**Figure 5:**
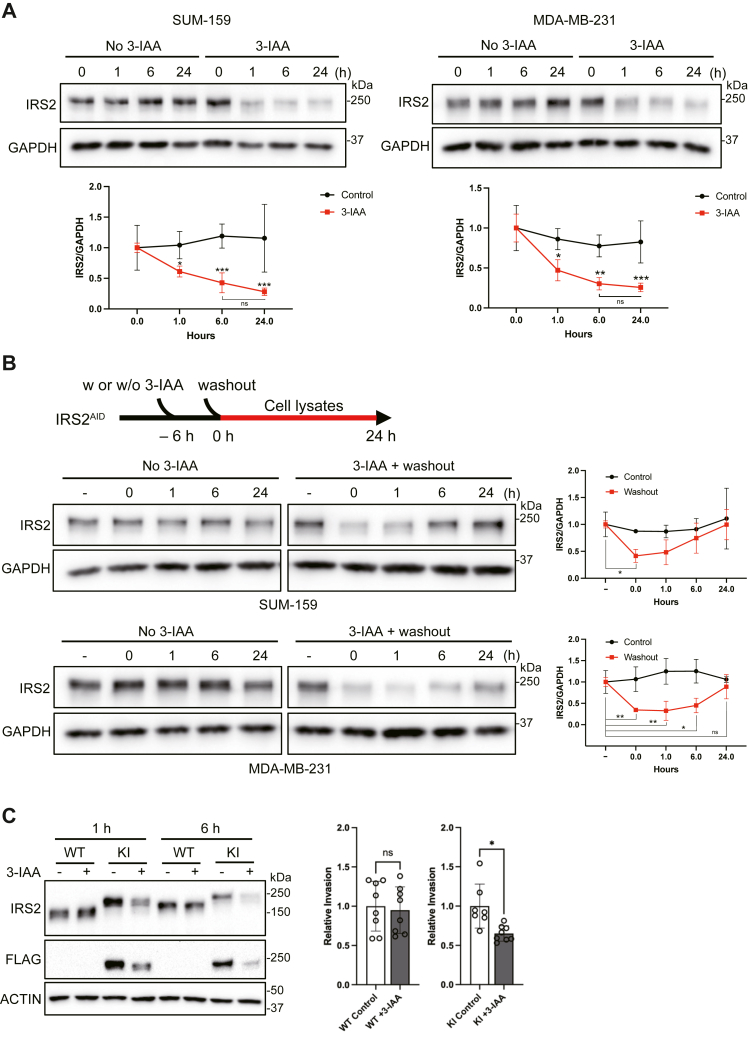
**Auxin-mediated degradation of DFN-IRS2.***A*, DFN-IRS2 (KI) cells were treated with or without 3-IAA (0.5 mM) for the indicated time periods and cell extracts were immunoblotted with IRS2 antibodies. The data shown in the graphs represent the mean ± S.D. of three independent experiments. *B*, DFN-IRS2 (KI) cells were treated with or without 0.5 mM 3-IAA for 6 h. The cells were then washed and allowed to recover for the indicated time periods in complete culture medium. The data shown in the graphs represent the mean ± S.D. of three independent experiments. *C*, parental (WT) and DFN-IRS2 (KI) SUM-159 cells were treated with 0.5 mM 3-IAA and assayed for invasion using Matrigel Transwell assays. The data shown represent the mean ± S.D. of invasion relative to untreated control cells from two independent experiments. Expression of DFN-IRS2 at the beginning and end of the assay is shown in the immunoblot. ∗*p* < 0.05, ∗∗*p* < 0.01, ∗∗∗*p* < 0.001.

Tagging IRS2 with an auxin-dependent degron allows for inducible and reversible suppression of endogenous IRS2 expression to study the impact of acute IRS2 loss on cellular function. To assess the efficiency of IRS2 degradation and recovery, DFN-IRS2 cells expressing the F-box protein *At*AFB2 were treated with the auxin derivative 3-IAA (500 μM). 3-IAA treatment induced the degradation of DFN-IRS2 in both SUM-159 and MDA-MB-231 KI cells in a time-dependent manner, with DFN-IRS2 levels significantly diminished after 6 h and reaching a plateau by 24 h (Fig. 5*A* and Fig. S1*C*). Upon wash-out of 3-IAA, DFN-IRS2 expression recovered in a time-dependent manner within 6 h (SUM-159) or 24 h (MDA-MB-231) (Fig. 5*B*). The recovery was faster in SUM-159 cells when compared with MDA-MB-231 cells, which could be due to differences in rates of *IRS2* transcription or translation in these cells. A low level of IRS2 expression remained in both cell lines after auxin treatment, which may reflect the inability of the F-box protein to access the AID tag on IRS2 due to either structural interference or spatial restriction of the two proteins.

A role for IRS2 in tumor cell invasion was established in our previous studies using cells chronically lacking IRS2 expression through Cre-lox recombination or CRISPR/Cas9-mediated gene knockout (10, 11). To evaluate the impact of acute reduction of IRS2 expression on tumor cell invasion, Transwell invasion assays were performed to evaluate invasive potential after exposure to auxin to induce DFN-IRS2 degradation (22). Parental (WT-IRS2) and DFN-IRS2 SUM-159 KI cells were pretreated with 3-IAA for 1 h prior to the start of the assays and 3-IAA was also included in the medium during the duration of the assays (5 h). Extracts from cells collected after the 1-h pre-treatment and at the end of the assay time (6 h total exposure) were analyzed by immunoblot to confirm suppression of DFN-IRS2 expression in the KI cells (Fig. 5*C*). Invasion diminished approximately 2-fold when IRS2 expression was acutely suppressed by auxin-mediated degradation in the DFN-IRS2 KI cells (Fig. 5*C*). Importantly, no change in WT-IRS2 expression or invasion was observed for the parental cells expressing WT-IRS2 when treated with 3-IAA, confirming that the invasive decrease of the DFN-IRS2 KI cells did not result from non-specific toxicity during the assay.

## Discussion

We used CRISPR/Cas9-mediated gene editing to modify endogenous IRS2 to study the expression, localization, and function of this adaptor protein. The introduction of an AID sequence at the N-terminus of the IRS2 protein provided rapid and reversible control of IRS2 protein degradation to investigate the impact of inducible reduction of IRS2 expression on cellular function. Our studies demonstrate that acute loss of IRS2 expression reduces tumor cell invasion, confirming the role of IRS2 in the regulation of invasive function that has been observed upon chronic knockout or knockdown of IRS2 (10, 11, 12). Knock-in of mNeon-green at the N-terminus facilitated the analysis of endogenous IRS2 expression and localization. Live fluorescence imaging of these cells revealed that IRS2 shuttles between the cytoplasm and nucleus, a function that had not been appreciated in previous studies. Specifically, IRS2 expression is primarily cytosolic when cells are grown in full growth medium, but it accumulates in the nucleus under growth deprivation conditions. PI3K signaling promotes the exclusion of IRS2 from the nucleus. A region in the IRS2 C-terminal tail that includes a predicted nuclear export signal is required for the export of IRS2 from the nucleus, as IRS2 accumulates in the nucleus when this region is deleted, and in the presence of the nuclear export inhibitor Leptomycin B. Together our data reveal new insight into IRS2 localization and highlight the value of studying endogenously tagged IRS2 as a model to examine IRS2 regulation and function.

Our studies of endogenous IRS2 tagged with mNeon-green provide new information about the regulation of IRS2 intracellular localization. In previous studies using fixed cells and indirect immunofluorescence, IRS2 expression was found to be primarily cytosolic (20). The absence of IRS2 nuclear localization was also observed in FFPE sections of human breast tumors and biochemical extraction of cells (8). Studying endogenous IRS2 expression in live cells without the introduction of potential artifacts from fixation or overexpression of exogenously tagged proteins has provided a more accurate assessment of the intracellular localization of this adaptor protein. In live cells imaged in a complete growth medium, IRS2 localization was observed to be primarily cytosolic. However, analysis of cells after serum starvation revealed a more dynamic regulation of IRS2 localization, with IRS2 nuclear accumulation correlating with growth deprivation conditions. These findings indicate that growth regulatory signals control the localization of IRS2 and our data support that PI3K signaling is one mechanism by which this occurs. The C-terminal region of IRS2 that contains the NES is highly disordered and the 3-dimensional structure of the protein is unknown. Post-translational modifications of IRS2, such as phosphorylation, may alter the exposure of the NES to facilitate or inhibit interactions with CRM1 and export from the nucleus. At this time, we cannot rule out the possibility that these modifications may also impact cytoplasmic retention or import. Inhibition of PI3K increases the mobility of IRS2 when resolved by SDS-PAGE, revealing a potential loss of phosphorylation that correlates with nuclear accumulation. This mechanism of regulating nucleo-cytoplasmic shuttling has been observed for other signaling proteins including PTEN and ERα (23, 24). How regulating IRS2 localization impacts its signaling functions remains to be determined. These mNeon-green knock-in cells will facilitate future real-time imaging studies to examine the regulatory mechanisms that control IRS2 localization and how this may impact signaling outcomes. Analysis of the DFN cells *in vivo* will also allow for the investigation of IRS2 expression and localization at the cellular and whole tumor levels.

The contributions of IRS2 to tumor cell biology have been revealed through studies using cell and mouse model systems in which the *IRS2* gene has been genetically knocked out by Cre-lox recombination or CRISPR/Cas9-mediated gene editing or knocked down by shRNA (1, 11, 12, 25). Through analysis of these models, a role for IRS2 in regulating breast cancer progression and metastasis has been revealed. IRS2 contributes to more aggressive tumor behavior through its ability to regulate CSC self-renewal to promote tumor initiation at both primary and metastatic sites and through the regulation of tumor cell invasion (1, 10, 11). However, the impact of acute loss of IRS2 function in established tumors, which would occur in the therapeutic setting, has not been assessed previously due to the absence of direct and specific targeted inhibitors of this adaptor protein. By introducing an auxin-inducible degron into the IRS2 protein, we have developed a model system that allows for the inducible degradation and restoration of IRS2 expression in a temporal manner. Our studies demonstrate that acute degradation of IRS2 recapitulates the reduction of tumor cell invasion that we previously observed upon chronic IRS2 loss, validating further investigation of how targeting IRS2 may impact tumor progression. These knock-in models that express IRS2 with both AID and mNeon-green tags will be useful both in the *in vitro* and *in vivo* setting to further explore IRS2 localization and function and its potential as a target for therapy.

## Experimental procedures

### Cells, antibodies, and reagents

SUM-159 cells were obtained from BioIVT and cultured in F-12 medium (Gibco) supplemented with 10% FBS, HEPES (25 mM), insulin (5 μg/ml), and hydrocortisone (1 μg/ml). MDA-MB-231 cells were obtained from ATCC and cultured in RPMI medium (Gibco) supplemented with 10% FBS. Cells were authenticated by STR profiling at the University of Arizona Genetics Core. All cells were incubated at 37 °C in 5% CO_2_. Cells were screened for *mycoplasma* contamination using the *Mycoplasma* PCR Detection Kit (Applied Biological Materials, Inc.). *IRS1/IRS2* double null SUM-159 cells and HA-tagged *WT-IRS2* and *IRS2* deletion mutants were generated and described previously (11, 12).

For auxin-induced degradation experiments, IAA Indole-3-acetic acid sodium (Sigma) was dissolved in NaOH (1 M) solution to a concentration of 500 mM, and cells were incubated with 0.5 mM 3-IAA prior to protein extraction. For washout experiments, cells were treated with 0.5 mM 3-IAA for 6 h, washed with PBS, and incubated in a complete culture medium before collection for immunoblot analysis. The *pSH-EFIRES-P-AtAFB2* plasmid was a gift from Elina Ikonen (Addgene plasmid #129715).

Primary antibodies: rabbit IRS2 (#4502; Cell Signaling Technology), rabbit mNeon-green (#55074; Cell Signaling Technology), mouse GAPDH (#sc-32233; Santa Cruz), mouse pTyr (sc-7020; Santa Cruz), rabbit p85 (#4292; Cell Signaling Technology), rabbit AKT (#9272; Cell Signaling Technology), rabbit pS473 AKT (#9271; Cell Signaling Technology), mouse FLAG (F1804; Sigma), rabbit HA (#3724; Cell Signaling Technology), mouse Lamin A/C (#sc-376248; Santa Cruz), mouse Actin (# MA5-11869; Thermo Fisher Scientific). Secondary antibodies: anti-rabbit IgG HRP (#111-035-144; Jackson ImmunoResearch), anti-mouse IgG HRP (#115-035-146; Jackson ImmunoResearch), anti-rabbit IgG AlexaFluor 594 (#A-11012; Invitrogen).

### Construction of gRNA and donor plasmid

gRNAs for *IRS2* were designed using the CRISPR tool implemented in CRISPOR (26). The N-terminal genomic region of the *IRS2* gene was targeted. The most efficient gRNA that cleaves within 20 bp of the start codon as measured by TIDE analysis was selected (Table 1) (27). *crRNA* and *tracrRNA* were purchased from IDT and made duplex for gRNA. A donor plasmid containing the *AID:3x-FLAG:mNeon-green:linker* nucleotide sequences (Table 1) flanked by left and right homology regions representing 789 bp upstream and 844 bp downstream of the *IRS2* start codon was generated by Azenta and used to target the N-terminal region of the *IRS2* gene by Cas9-mediated HDR. The *IRS2* start codon was removed from the genomic sequence of the right homology arm. A donor fragment was generated from the plasmid template by PCR using Powerpol 2× PCR mix (ABclonal) (primer sequences, Table 1). The parameters were as follows: 98 °C for 45 s, then 30 cycles of 98 °C for 10 s, 62.8 °C for 30 s, 72 °C for 90 s (30 s/kb), and a final step at 72 °C for 5 min.

**Table 1:** gRNA, Primer and Donor sequences

gRNA, primer or donor	Sequence (5′−3′)
gRNA	
*IRS2* gRNA	CCGCGATGGCGAGCCCGCCG
*AAVS1* gRNA	GGGGCCACTAGGGACAGGAT
Primer	
donor fragment	
Forward	TAATTGAGTCCGAGGCGGGA
Reverse	TCCAGGATGGTCTCGTGGAT
genotype (1st)	
Forward	GCATCCTCAGGAGCCCCA
Reverse	ATGTTCAGGCAGCAGTCGAG
genotype (2nd)	
Forward	CCGCCCTCGCCCAAAAT
Reverse	GCTCCCAGCCACCGAC
Donor	
*miniIAA7*	AAGAGGGGCTTCTCTGAGACCGTGGACCTGATGCTGAACCTGCAGTCCAATAAGGAGGGCTCTGTGGATCTGAAGAACGTGAGCGCCGTGCCTAAGGAGAAGACCACACTGAAGGACCCATCCAAGCCCCCTGCCAAGGCACAGGTGGTGGGATGGCCACCCGTGCGGAACTACAGAAAGAATATGATGACCCAGCAGAAGACAAGCTCCGGCGCAGAGGAGGCATCTAGCGAGAAGGCCGGCAATTTTGGAGGAGGAGCAGCAGGAGCAGGACTGGTGAAGGTGTCCATGGACGGAGCACCATACCTGCGGAAGGTGGATCTGAAGATGTATAAG
*3×-FLAG*	GACTACAAAGACCATGACGGTGATTATAAAGATCATGACATCGATTACAAGGATGACGATGACAAG
*mNeon-green*	GTGAGCAAGGGCGAGGAGGATAACATGGCCTCTCTCCCAGCGACACATGAGTTACACATCTTTGGCTCCATCAACGGTGTGGACTTTGACATGGTGGGTCAGGGCACCGGCAATCCAAATGATGGTTATGAGGAGTTAAACCTGAAGTCCACCAAGGGTGACCTCCAGTTCTCCCCCTGGATTCTGGTCCCTCATATCGGGTATGGCTTCCATCAGTACCTGCCCTACCCTGACGGGATGTCGCCTTTCCAGGCCGCCATGGTAGATGGCTCCGGATACCAAGTCCATCGCACAATGCAGTTTGAAGATGGTGCCTCCCTTACTGTTAACTACCGCTACACCTACGAGGGAAGCCACATCAAAGGAGAGGCCCAGGTGAAGGGGACTGGTTTCCCTGCTGACGGTCCTGTGATGACCAACTCGCTGACCGCTGCGGACTGGTGCAGGTCGAAGAAGACTTACCCCAACGACAAAACCATCATCAGTACCTTTAAGTGGAGTTACACCACTGGAAATGGCAAGCGCTACCGGAGCACTGCGCGGACCACCTACACCTTTGCCAAGCCAATGGCGGCTAACTATCTGAAGAACCAGCCGATGTACGTGTTCCGTAAGACGGAGCTCAAGCACTCCAAGACCGAGCTCAACTTCAAGGAGTGGCAAAAGGCCTTTACCGATGTGATGGGCATGGACGAGCTGTACAAG
*Linker*	GGTGGTGGCGGTTCAGGCGGAGGTGGCTCT

### Generation of IRS2 knock-in cells

1.0 × 10^6^ cells were electroporated with 240 pmol crRNA: tracrRNA duplex,103.7 pmol S.p. Cas9 Nuclease V3 (IDT), and 3.0 μg donor fragment per 100 μl of Nucleofector Solution V (Lonza) using the Amaxa Nucleofector II (Lonza). For MDA-MB-231 cells, 60 pmol Cas9 Electroporation enhancer (IDT) was included. After electroporation, 500 μl of prewarmed culture media was added per cuvette and the cells were gently resuspended and transferred to a 6-well plate with 2 ml culture media (SUM-159) and 1.17 pmol HDR enhancer V2 (IDT) (MDA-MB-231). Cells were incubated for 24 h (SUM-159) or 48 h (MDA-MB-231) prior to sorting mNeon-green positive cells into 96 well plates using the FACSMelody (BD Biosciences). Single-cell clones were expanded and screened by PCR for homozygous knock-in of the DFN cassette.

Homozygously tagged clones (DFN-IRS2 KI cells) were used to express the auxin receptor F-box protein *At*AFB2 in the *AAVS1* safe harbor locus. 1.0 × 10^6^ cells were electroporated as above with 3.0 μg pSH-EFIRES-P-AtAFB2 plasmid (16), gRNA (28) and Cas9. Electroporated cells were plated into 10 cm plates and incubated for 24 h prior to selection with 1 μg/ml puromycin.

### Genotyping PCR

Cells were lysed in QuickExtract DNA Extraction Soln 1.0 (BIOSEARCH Technologies) for 15 min at 68 °C and 15 min at 98°C. Target regions were amplified by PCR using Powerpol 2× PCR mix (Table 1). The parameters were as follows: 98 °C for 45 s, then 30 cycles of 98 °C for 10 s, 71 °C for 30 s, 72 °C for 1 min (30 s/kb), and a final step at 72°C for 5 min for focused on tag region (first round PCR) and 98°C for 45 s, then 35 cycles of 98°C for 10 s, 66°C for 30 s, 72 °C for 2 min (30 s/kb), and a final step at 72 °C for 5 min for over insert region (second round PCR). The amplified products were analyzed by agarose gel electrophoresis.

### Immunoprecipitation and immunoblotting

Cells were seeded in plates and grown for 24 h before protein extraction in lysis buffer (20 mM Tris-HCl pH 7.4, 137 mM NaCl, 10% Glycerol, and 1% NP-40) containing 1× complete Mini (Roche) and 1× PhosSTOP (Roche). Lysates were centrifuged at 13,000 rpm for 10 min at 4°C and the protein concentrations were quantified using Pierce BCA Protein Assay Kit (Thermo Fisher). For immunoprecipitations, aliquots of cell extracts containing equivalent amounts of protein were pre-cleared for 1 h with non-specific IgG and protein-A beads (GE Healthcare) and then incubated for 3 h with specific antibodies and protein-A beads with constant agitation. The beads were washed three times in extraction buffer and laemmli sample buffer was added to the samples. Whole-cell extracts containing equivalent amounts of protein or immune complexes were separated by 8% SDS-PAGE and then transferred onto nitrocellulose membranes (Thermo Fisher). After blocking in 5% milk/TBST at room temperature for 1 h, the membranes were incubated with primary antibodies at 4 °C overnight and subsequently incubated with HRP-conjugated secondary antibodies at room temperature for 1 h. Proteins were detected by enhanced chemiluminescence, and all images were acquired using a ChemiDoc XRS + system (Bio-Rad). GAPDH and Actin antibodies were used at a 1:2000 dilution with TBST containing 3% BSA (Sigma), and other primary antibodies were used at a 1:1000 dilution. All secondary antibodies were used at a 1:5000 dilution with TBST containing 3% BSA. Densitometry was performed using ImageJ.

### Fluorescence microscopy

For live cell imaging, cells were plated in μ-slide 8 well high glass bottom (SUM-159) or μ-slide 8 well high ibiTreat (MDA-MB-231) chambers (ibidi) and incubated in either complete culture medium or medium lacking FBS (serum-starved) and then viewed by confocal microscopy (Nikon ECLIPSE Ti2;20×; 37 °C with 5% CO_2_). For nuclear export inhibition, cells were incubated in 20 nM Leptomycin B prior to imaging. For fixed cell imaging, subconfluent cells in 8-well chamber glass slides grown in complete culture medium were washed with PBS and fixed in 3.8% paraformaldehyde in PBS for 15 min at room temperature. Fixed cells were blocked for 1 h using PBS + 5% FBS + 0.3% Triton X-100 and then incubated with anti-HA antibodies diluted in PBS + 1% BSA + 0.1% Triton X-100 overnight at 4 °C. Cells were incubated in secondary antibodies and DAPI diluted in the same buffer for 2 h at room temperature. Cells were washed three times with PBS after both primary and secondary antibody incubations, a mounting medium was added, and cells were viewed by fluorescence microscopy (Zeiss AxioObserver; 20×). Images were adjusted equally for brightness/contrast to measure IRS2 intensity in the nucleus and cytoplasm using ImageJ software and are pseudocolored green.

### Cytoplasmic and nuclear extraction

Nuclear and cytoplasmic fractions were extracted using the NE-PER Nuclear and Cytoplasmic Extraction Reagents Kit (Cat. # 78833, Thermo Fisher Scientific) following the manufacturer's instructions. Briefly, cells were lysed in ice-cold CER I buffer with protease inhibitor. After 10 min on ice, CER II buffer was added in a volume ratio of 200:11 (CER I:CER II). The lysate was centrifuged at 13,000 rpm for 5 min, and the cytoplasmic extract (supernatant) was transferred to a clean tube. The nuclear pellet was resuspended in ice-cold NER buffer, vortexed for 15 s every 10 min for 40 min, and then centrifuged at 13,000 rpm for 10 min. The resulting nuclear extract (supernatant) was transferred to a clean tube. To inhibit PI3K signaling, cells were incubated with BKM120 (5 μM) or vehicle (DMSO) for 3 h prior to extraction.

### Matrigel invasion assay

Matrigel Transwell invasion assays were performed using 6.5 mm Transwell chambers (8 μm pore size) as described previously (22). Cells were pre-incubated with IAA Indole-3-acetic acid sodium at a concentration of 0.5 mM for 1 h prior to the assays and the drug was also present in the upper and lower wells of the Transwell chamber during the assays. After 5 h, the cells that had invaded to the lower surface of the filters were fixed in methanol for 10 min. The fixed membranes were mounted on glass slides using a Vectashield mounting medium containing 4′,6-diamidino-2-phenylindole (Vector Laboratories, Burlingame, CA). Invasion was quantified by counting the number of stained nuclei in five independent fields in each Transwell.

### Data analysis

Statistical analysis was performed using Prism10.0, Graphpad. Data were analyzed using unpaired *t* test with Welch's correction between two groups (Figs. 3, *A* and *C*, and 5*C*), paired *t* test between two groups (Fig. 3*B*), ordinary One-way ANOVA (Figs. 3*D* and 4, *C* and *D*), RM One-way ANOVA (Fig. 4*B*), and two-way ANOVA with Dunnett’s post-test (Fig. 5, *A* and *B*). *p*-value of <0.05 was considered to indicate statistical significance.

## Data availability

All data are contained within the manuscript.

## Supporting information

This article contains supporting information.

## Conflict of interest

The authors declare that they have no conflicts of interest with the contents of this article.
