# Supplementary material for: Fluorescent tagging of endogenous IRS2 with an auxin-dependent degron to assess dynamic intracellular localization and function
Source: J Biol Chem. 2024 Sep 19;300(11):107796. doi: 10.1016/j.jbc.2024.107796 (PMC11513485; doi:10.1016/j.jbc.2024.107796)
Supplement: Supplementary Figure 1 [file mmc4.pdf]

Supplemental Figure 1

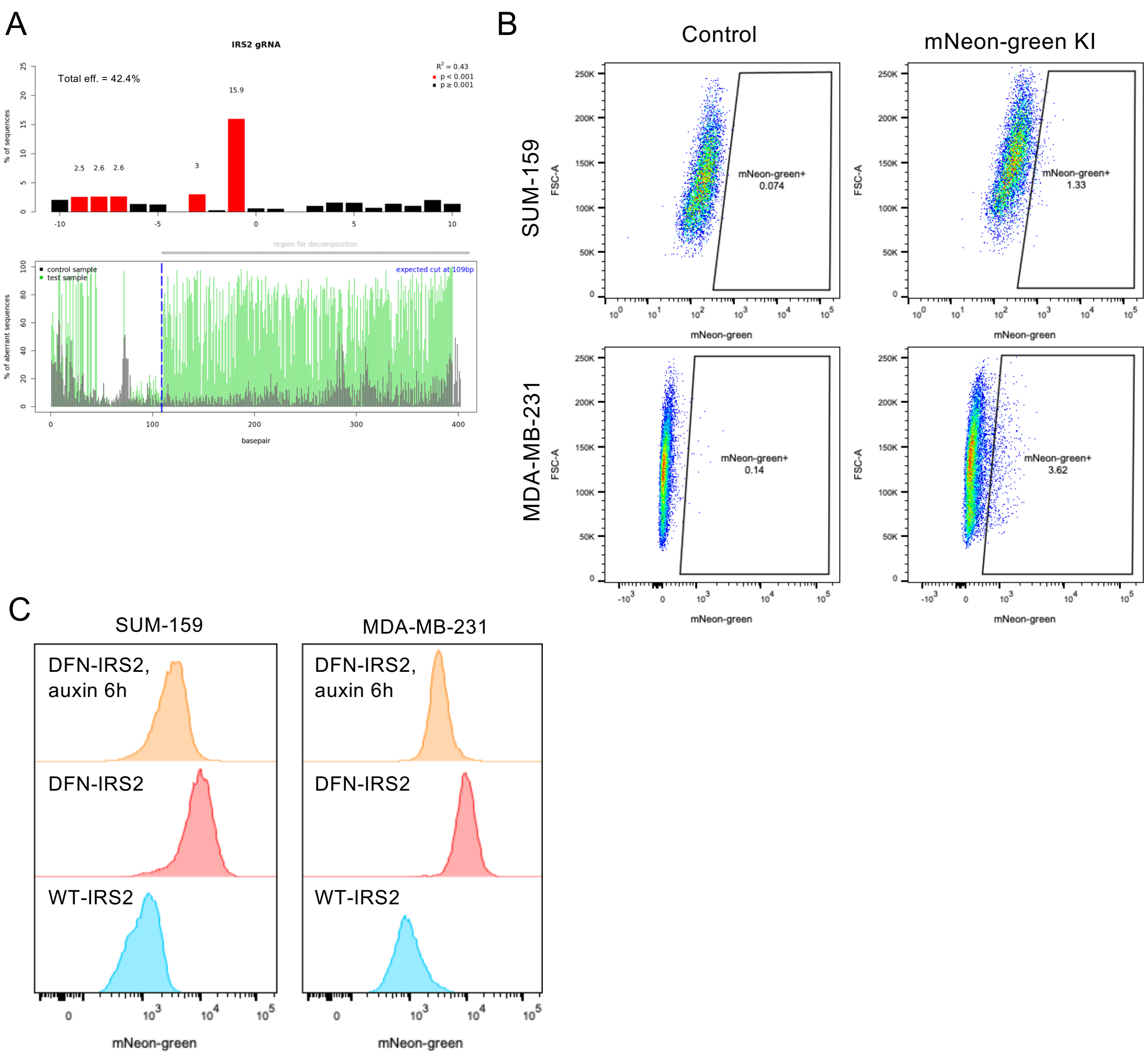

Supplemental Figure 1. Generation and characterization of DFN-KI cells. A. TIDE analysis for the gRNA used to knock-in the DFN cassette by homology directed repair. B. Density plots for the sorting of mNeon-green positive cells. C. Flow cytometry analysis for mNeon green in WT-IRS2 cells, DFN-IRS2 cells and DFN-IRS2 cells after a 6 hrs treatment with auxin.
